# The Economic Burden and Impact on Quality of Life of Herpes Zoster and Postherpetic Neuralgia in Individuals Aged 50 Years or Older in Italy

**DOI:** 10.1093/ofid/ofz007

**Published:** 2019-01-12

**Authors:** Sean Matthews, Antonio De Maria, Marco Passamonti, Giovanni Ristori, Idalba Loiacono, Anna Puggina, Desmond Curran

**Affiliations:** 1 Value Evidence, GSK, Wavre, Belgium; 2 Investigator site, Puglia, Galatina, Italy; 3 Investigator site, Lombardia, Fagnano Olona, Italy; 4 Investigator site, Lazio, Roma, Italy; 5 Payers and Evidence Solutions, GSK, Verona, Italy

**Keywords:** herpes zoster (HZ), postherpetic neuralgia (PHN), quality of life (QoL), QALY; economic burden

## Abstract

**Background:**

To estimate the health care resource utilization, costs, and impact on quality of life (QoL) of herpes zoster (HZ) and postherpetic neuralgia (PHN) in adults aged ≥50 years in Italy.

**Methods:**

This was a prospective, observational, multicenter, community physician–based surveillance study (NCT01772160) in Italy. Health-related QoL data were collected using the EuroQoL-5 Dimension (EQ-5D) and Zoster Brief Pain Inventory (ZBPI) questionnaires. Both questionnaires were assessed at days 0 (HZ rash onset), 15, 30, 60, and 90 for all patients, and monthly thereafter for patients who developed PHN. Resource utilization was recorded for 3 months post–HZ onset and 9 months for PHN patients. Costs from both payer and societal perspectives were estimated and were composed of direct medical costs (general practitioner/specialist visits, procedures, hospitalizations, medications), work loss by patient/caregiver, and transport costs.

**Results:**

A total of 391 patients with HZ were included, of whom 40 developed PHN. The mean ZBPI worst pain score was 5.7 at day 0, reducing to 2.6 at day 30 and 0.7 by day 90. Patients with PHN had a mean worst pain score of 5.7 at day 90. We estimated an overall disutility associated with HZ of 0.134. The mean cost per HZ patient from a payer/societal perspective was €153/€298, respectively, and the mean cost per HZ patients who developed PHN was €176/€426, respectively.

**Conclusions:**

HZ is associated with impaired QoL and substantial health care resource use, highlighting the need for preventive strategies. This could reduce the disease burden for the patient and health care system.

**ClinicalTrials.gov study registry:**

NCT01772160.

Herpes zoster (HZ), or “shingles,” is a viral disease characterized by a painful skin rash that results from the reactivation of latent varicella zoster virus from the dorsal root or cranial nerve ganglia. It has been observed that the lifetime risk of developing HZ is about 20%–30% [1–3]. It is well documented that incidence of HZ increases with age, particularly from the age of 50 years [4]. The most common complication of HZ is zoster-associated pain, which may be classified as acute, subacute herpetic pain, and postherpetic neuralgia (PHN), a chronic pain that persists or develops after 90 days from HZ rash onset.

Data on the burden of disease and the impact of HZ and PHN on Italian patients’ quality of life (QoL) are somewhat scarce and mostly limited to retrospective studies. A prospective study was carried out in 2010 in adults aged ≥50 years across Italy that demonstrated that HZ and PHN represent an important burden of disease in the elderly [5]. There is a particular lack of accurate utility loss data associated with health-related QoL caused by HZ and PHN specific to Italy.

Accurate data on the economic burden of HZ and PHN are also somewhat scarce. A prospective study was carried out in 2004 in Piemonte to estimate the incidence and costs associated with HZ in patients >14 years of age [6], and a retrospective study focused primarily on immunocompetent patients aged ≥50 years across 4 regions: Veneto, Toscana, Lazio, and Campania [7]. Levi et al. conducted a retrospective search of hospital discharge records between 2002 and 2012 in Tuscany [8], and more recently a cross-sectional study on hospitalizations related to HZ during the period 2008–2016 in the Veneto region was published [9].

The aim of our prospective study was thus to address the lack of existing information related to the clinical and economic burden as well as the impact on QoL in order to aid and improve public health care decisions related to HZ prevention in Italy. A prospective surveillance of HZ cases, starting at the diagnosis of the acute HZ episode, represents a suitable and optimal approach to describe the clinical and economic burden and effect on QoL.

## METHODS

This prospective, observational, multicenter community-based study was carried out in primary care practices in northern Italy from 2013 to 2016 (ClinicalTrials.gov Identifier: NCT01772160).

Participating physicians were general practitioners (GPs) affiliated with the Italian Society of GPs (ie, SIMG) and belonging to 9 different networks taking part in the study; a network, in this case, is defined as a group of GPs located in the same town/province and linked to the same Local Health Unit/ethics committee. The 9 networks were located in the following regions: Lombardia, Piemonte, Toscana, Umbria, Lazio, Campania, and Puglia, covering an estimated 44 000 persons aged ≥50 years.

Adults 50 years of age or older presenting with HZ were invited to participate in the study, which aimed to estimate the HZ incidence rate, the proportion of HZ patients developing PHN, and the economic burden and impact on health-related QoL of both HZ and PHN. The analysis of the incidence of HZ and PHN will be presented in a separate manuscript. All patients were to be followed up until assessment on day 90 after the initial visit, which corresponded to the first time the patient presented at the study site (Supplementary Data). Patients who developed PHN were invited to continue in the study until assessment at day 180. Patients who were still experiencing pain at day 180 were invited to continue in the study until day 270.

Each patient received a booklet containing the EuroQoL-5 Dimension (EQ-5D) and Zoster Brief Pain Inventory (ZBPI) questionnaires, which were to be completed at days 0, 15, 30, 60, and 90. For those patients still in the study beyond day 90, both questionnaires were to be collected at days 120, 150, 180, 210, 240, and 270.

Resource utilization related to the HZ episode was recorded by the GP at each visit. Resources recorded included GP and specialist visits, hospital outpatient visits and admissions, procedures and tests performed, and medications taken. Mode of transportation to study sites was also collected, as were details on work loss due to the HZ episode for participants or for their caregivers.

### Pain Assessment and QoL

The ZBPI questionnaire was used to quantify HZ pain and discomfort and to measure the impact on activities of daily living (ADL) and health. The ZBPI questionnaire was adapted from the Brief Pain Inventory to make it an HZ-specific measure of pain severity that captures pain and discomfort caused by HZ (Supplementary Data) [10].

Quality of life was assessed by the standardized, generic EQ-5D questionnaire [11, 12]. A summary weighted health utility score was calculated using country-specific weights derived from a sample of the general population (in this case, Italian time-trade-off values were used [13]). The “worst health” state imaginable corresponds to a utility score of –0.38, whereas 1.00 represents “perfect health” (Supplementary Data).

In this analysis, questionnaires were assigned to time points based on the completion date relative to the rash onset date (Supplementary Data and Supplementary Table 1).

### Costs and Resource Utilization

Unit costs for GP and specialist visits, procedures, and medications were taken from official sources and are detailed in Supplementary Table 2. As the study was carried out in a primary care setting, some hospitalizations and all specialist visits were only reported as referrals. As a consequence, no details of length of hospital stay were recorded, so a mean of 7.8 days and a total cost of €2695 per stay were assumed [14, 15].

Patients were also required to indicate their mode of transport when traveling to the healthcare provider. The cost of both private and public transport was calculated based on the total number of kilometers traveled. A unit cost was assigned per kilometer to obtain total costs, as detailed in Supplementary Table 2.

Working days lost due to the HZ episode for both the patient and the caregiver were valued by multiplying the number of days recorded by the national average daily earnings, as defined by the most recently available national labor statistics (Supplementary Table 2).

Costs were calculated from both the payer (ie, health care system) and the societal perspective. All direct medical costs are fully reimbursed by the health care system, with the exception of medications that are only partly reimbursed. Costs from the societal perspective are composed of all direct and indirect costs.

## RESULTS

### Demographics

The demographics of the 391 HZ patients enrolled in the study are presented in Table 1. The mean (SD) age was 69.3 (10.95) years. Overall, 39.4% were male and 60.6% female. In total, 67 patients (17.1%) were still working, 61 of whom were younger than 65 years of age (YOA).

**Table 1.** Patient Demographics by Age Group and Overall

		50–59 YOA (n = 87)		60–64 YOA (n = 38)		65–69 YOA (n = 88)		70–79 YOA (n = 98)		≥80 YOA (n = 80)		Overall ≥50 YOA (n = 391)	
		n	%	n	%	n	%	n	%	n	%	n	%
Gender	Male	22	25.3	20	52.6	38	43.2	43	43.9	31	38.8	154	39.4
	Female	65	74.7	18	47.4	50	56.8	55	56.1	49	61.3	237	60.6
Employment	Self-employed	52	59.8	9	23.7	4	4.5	2	2.0	0	-	67	17.1
	Unemployed	13	14.9	6	15.8	1	1.1	0	-	0	-	20	5.1
	Retired	8	9.2	20	52.6	77	87.5	91	92.9	71	88.8	267	68.3
	Other (unpaid)	13	14.9	3	7.9	6	6.8	5	5.1	9	11.3	36	9.2
	Missing	1	1.1	0	-	0	-	0	-	0	-	1	0.3
Age, y	Mean	54.5		62.2		67.1		74.4		85.1		69.3	
	SD	2.68		1.50		1.37		2.69		4.10		10.95	
	Median	55.0		62.5		67.0		74.0		84.0		69	
	Min, Max	50, 59		60, 64		65, 69		70, 79		80, 95		50, 95	

Abbreviations: HZ, herpes zoster; min, minimum value; max, maximum value; YOA, years of age.

### Questionnaire Compliance

Of the 391 patients, 388 returned at least 1 evaluable ZBPI and EQ-5D questionnaire. A total of 343 patients returned at least one evaluable ZBPI questionnaire on or after 90 days following rash onset and were thus evaluable for PHN assessment. The number of patients who developed PHN and thus continued in the study beyond day 90 was 40 (10.2% of all enrolled).

The rate of completion of evaluable questionnaires was very high throughout the initial assessment period up to day 90, with compliance >90% at all time points. For the 40 PHN patients continuing in follow-up, a drop in the completion rate was seen after day 90, with 16 patients (40%) completing the assessment at day 120. Only 6 patients continued in the study after day 180.

### ZBPI Questionnaire

**Figure 1.**
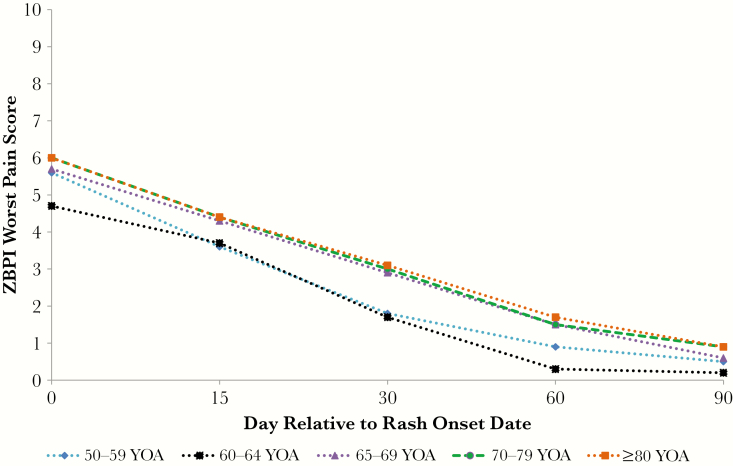
Mean Zoster Brief Pain Inventory worst pain scores by age category and time. Abbreviations: YOA, years of age; ZBPI, Zoster Brief Pain Inventory.

At day 0, the overall mean (SD) ZBPI worst pain score was 5.7 (2.63), reducing to 2.6 (3.04) at day 30 and 0.7 (1.94) at day 90. The mean ZBPI worst pain score was similar across age groups at day 0 (Figure 1). At subsequent time points, patients under the age of 65 appeared to have a lower mean worst pain score than those older than 65 years.

The mean ZBPI worst pain scores categorized by patients who developed PHN and patients who experienced HZ only are presented in Supplementary Figure 1. The mean (SD) score at day 0 for PHN patients was 7.8 (2.18), which reduced to 5.7 (1.96) at day 90 (Supplementary Table 5).

The mean (SD) ZBPI ADL score for all enrolled patients was 3.48 (2.71) at day 0 and diminished monotonically over time to 1.82 (2.46) at day 30 and 0.58 (1.47) at day 90. The HZ episode appeared to have a greater impact on patients in the ≥80 YOA group, in which the mean (SD) score of 4.49 (2.86) at day 0 had reduced to 2.35 (2.67) at day 30 (Supplementary Table 6). The impact on ADL generally increased with age. The individual ADL items most affected were sleep and mood, with a mean score (SD) of 4.1 (3.39) and 4.3 (3.33), respectively, at day 0, whereas walking ability, with a day 0 mean (SD) of 1.9 (2.73), was the least affected (Supplementary Figure 2).

### Analysis of QoL Using the EQ-5D

The estimated EQ-5D utility score least-squares (LS) means by age group over time are presented in Table 2. The estimated LS mean (SE) at day 0 was 0.746 (0.011), which increased (improved) to 0.848 (0.011) by day 30. We estimated from the longitudinal model an overall LS mean (SE) of 0.798 (0.009) over the 30 days after rash onset for all enrolled patients, and we also estimated that the LS mean (SE) utility for PHN patients for the period days 90–120 was 0.717 (0.037). By assuming that the mean score at day 90 reflects a normal or baseline utility, we estimated a mean disutility of 0.134 for all patients for the first 30 days of the HZ episode, which represents a quality-adjusted life-year (QALY) loss of 0.011 per month. The highest disutility of 0.150 (QALY loss, 0.012 per month) was observed in the ≥80 YOA group. We similarly calculated a mean disutility of 0.131 for patients with HZ only (ie, those who did not develop PHN) for the first 30 days of the HZ episode.

**Table 2.** Estimated EQ-5D Least-Squares Mean Utility Scores by Time and Age Group

	50–59 YOA (n = 87)		60–64 YOA (n = 38)		65–69 YOA (n = 88)		70–79 YOA (n = 98)		≥ 80 YOA (n = 80)		Overall ≥50 YOA (n = 391)	
Day	Mean	SE	Mean	SE	Mean	SE	Mean	SE	Mean	SE	Mean	SE
0	0.767	0.023	0.807	0.035	0.769	0.022	0.738	0.021	0.677	0.023	0.746	0.011
15	0.853	0.022	0.855	0.031	0.810	0.021	0.792	0.020	0.723	0.022	0.801	0.010
30	0.902	0.022	0.914	0.033	0.851	0.022	0.833	0.021	0.778	0.023	0.848	0.011
60	0.951	0.019	0.927	0.028	0.926	0.019	0.897	0.017	0.809	0.019	0.900	0.009
90	0.969	0.016	0.970	0.024	0.939	0.016	0.925	0.015	0.876	0.017	0.932	0.008
0–30^a^	0.841	0.019	0.859	0.028	0.810	0.018	0.788	0.017	0.726	0.019	0.798	0.009
Disutility^b^	0.128	-	0.111	-	0.129	-	0.137	-	0.150	-	0.134	-
QALY^c^	0.011		0.009		0.011		0.011		0.012		0.011	
HZ-only patients^d^												
90	0.974	0.014	0.987	0.020	0.969	0.014	0.953	0.014	0.902	0.015	0.955	0.007
0–30	0.855	0.017	0.871	0.025	0.845	0.017	0.819	0.016	0.746	0.018	0.824	0.008
Disutility	0.119	-	0.106	-	0.124	-	0.134	-	0.156	-	0.131	
QALY	0.010		0.009		0.010		0.011		0.013		0.011	

Abbreviations: EQ-5D, EuroQoL-5 Dimension; HZ, herpes zoster; QALY, quality-adjusted life-year; YOA, years of age.

^a^0–30 is the estimated mean utility score over days 0–30.

^b^In the disutility score calculation, the estimated mean values at day 90 serve as the normal or baseline utility score.

^c^Calculated as disutility value multiplied by (length of period in days)/(number of days in year); ie, 30/365.

^d^HZ-only patients; all patients who did not develop postherpetic neuralgia.

### Direct Medical Resource Utilization and Costs

In total, 853 outpatient appointments were observed for the 391 enrolled patients, a mean of approximately 2.2 visits per patient. In total, 7 patients were hospitalized (1.8%) and 2 patients had an emergency room visit. A total of 53 specialist visits were recorded for 38 patients (9.7%), and 20 patients (5.1%) had a total of 23 ophthalmologist visits.

The most commonly observed diagnostic or therapeutic procedures were blood collection for related tests and dressing of the area with rash, which were observed in 4.1% and 3.3% of the enrolled patients, respectively.

Supplementary Table 3 presents the most commonly prescribed medications by anatomical therapeutic chemical (ATC) level 2 category and age group. A total of 365 (93.4%) patients were prescribed antivirals for systemic use. The most common medications in this category were valaciclovir (32.7% of patients), brivudina (30.2%), and aciclovir (25.6%). A total of 127 patients (32.5%) were prescribed antibiotics and chemotherapeutics for dermatological use, of which the most common medication was aciclovir (113 patients, 28.9%). Antiepileptics were prescribed for 119 patients (30.4%), of which the most common medication was pregabalin (107 patients, 27.4%).

Table 3 presents the direct medical costs by payer perspective, cost category, and age group. All of the direct costs were the same from a payer and societal perspective, except for the costs related to medications. Two patients had an emergency room visit at a cost of €241 per patient. The overall mean cost of GP visits was €24 per patient. The number of GP visits and associated mean costs increased with age. The overall mean cost of specialist visits was €3 per patient. The overall mean societal cost of medications was €112 per patient. The mean cost of procedures/lab tests was €2 per patient. A cost of €2695 was assumed for each of the 7 patients who were hospitalized. Combining these components gives an overall mean (SD) direct medical cost of €153 (€368) from a payer perspective and €189 (€377) from a societal perspective per enrolled patient.

**Table 3.** Direct Medical Costs by Component and Total Costs by Complication Category by Analytical Perspective and Age Group

		50–59 YOA (n = 87)		60–64 YOA (n = 38)		65–69 YOA (n = 88)		70–79 YOA (n = 98)		≥80 YOA (n = 80)		Overall ≥50 YOA (n = 391)	
	Perspective	n	Mean, €	n	Mean, €	n	Mean, €	n	Mean, €	n	Mean, €	n	Mean, €
Direct medical costs													
Emergency room visits	HCS/soc	1	241	-	-	-	-	1	241	-	-	2	241
GP visits	HCS/soc	87	20	38	21	88	25	98	27	80	27	391	24
Medications	HCS	86	70	38	58	87	72	97	87	80	76	388	75
	Soc	86	101	38	84	87	105	97	131	80	120	388	112
Procedures/lab tests	HCS/soc	6	24	2	25	6	23	12	29	5	26	31	26
Specialist visits	HCS/soc	7	24	3	21	12	34	14	30	2	21	38	29
Hospital admissions	HCS/soc	1	2695	1	2695	2	2695	3	2695	-	-	7	2695
Overall mean^a^	HCS	87	126	38	153	88	164	98	206	80	105	391	153
	Soc	87	157	38	179	88	195	98	249	80	149	391	189
Total costs by complication category													
HZ only	HCS	66	133	27	72	60	86	62	129	53	96	268	108
	Soc	66	331	27	113	60	113	62	179	53	139	268	187^b^
PHN	HCS	6	94	1	117	9	128	12	299	12	135	40	176
	Soc	6	1164	1	186	9	207	12	429	12	239	40	426^b^
HZ with complications (excluding PHN)	HCS	15	107	10	373	19	425	24	359	15	114	83	286
	Soc	15	1067	10	990	19	500	24	459	15	181	83	592^b^
All HZ cases	HCS	87	126	38	153	88	164	98	206	80	105	391	153
	Soc	87	515	38	346	88	206	98	278	80	162	391	297^b^

Abbreviations: GP, general practitioner; HCS, health care system; HZ, herpes zoster; mean, mean cost in € averaging only over the patients generating this cost component; PHN, postherpetic neuralgia; soc, societal; spec, specialist; YOA, years of age.

^a^The mean cost in € by averaging each cost component over all 391 patients and adding to all the cost components.

^b^Indirect costs due to work absence only occurred for patients still working but were averaged over all 391 patients to estimate the average total societal cost per HZ patient (and in each HZ category).

### Direct Nonmedical Costs

Patients traveled an average of 4.6 km to the health care provider. The most frequent mode of transport was private car, used by 257 patients. One hundred thirty-nine patients were able to walk to their health care provider, and 4 patients required an ambulance. The mean combined transport cost was €13 per patient.

### Indirect Costs

A total of 16 HZ patients detailed lost work days (14 females and 2 males). A median of 15.5 days of work was lost for these patients. All patients who missed work were under 65 years of age. In total, 12 caregivers were reported to have lost working days. Of the 12 caregivers, 6 were paid and 6 were unpaid. All of the 6 caregivers in paid employment were associated with HZ patients over the age of 65, and 3 were for HZ patients ≥80.

The mean costs of work losses from a societal perspective were €349 per patient aged 50–59 (n = 87) and €152 per patient aged 60–64 (n = 38).

### Combined Costs

Table 3 presents the total costs by complication category and overall. The mean cost (SD) of an HZ episode per patient was €153 (€368) from a payer perspective and €297 (€730) from a societal perspective. The highest costs from a societal perspective were observed in the 50–59 YOA group (mean, €515). Direct medical costs represented 63.6% of costs, patient work loss 31.0%, caregiver work loss 1.0%, and transport costs 4.4% (Supplementary Figure 3).

The mean societal cost per male patient is €192, compared with €366 per female patient. The mean costs for patients with PHN were €176 and €426 from the payer and societal perspectives, respectively (Supplementary Table 7).

**Figure 2.**
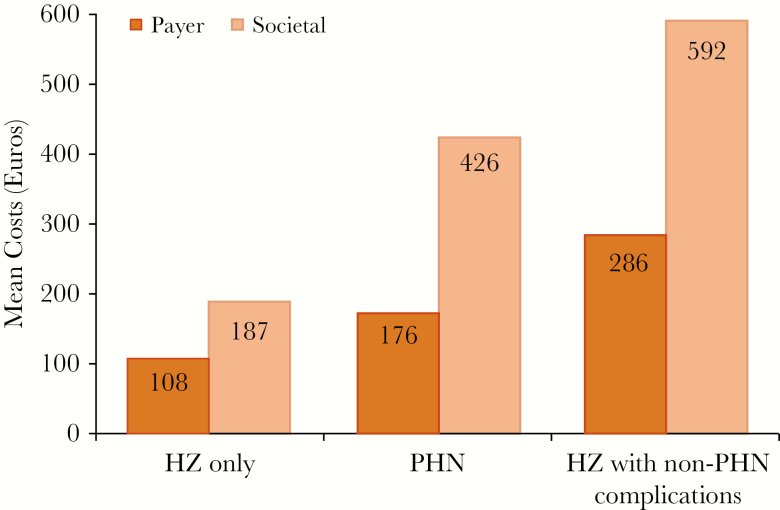
Mean costs by payer perspective and complication category. Abbreviations: HZ, herpes zoster; PHN, postherpetic neuralgia.

The highest costs were observed in patients who experienced non-PHN complications, with mean costs per patient of €286 and €592 from the payer and societal perspectives, respectively (Figure 2). Within this category, the highest costs were experienced by the 15 patients with ocular complications, with mean costs of €669 and €1582 from the payer and societal perspectives, respectively (Supplementary Table 4).

## DISCUSSION

This prospective, GP practice–based cohort study is one of the first to estimate both the economic burden and impact on QoL of HZ and PHN in Italian adults ≥50 YOA.

We estimated that HZ patients had a mean ZBPI worst pain score of 5.7 at day 0, which reduced to 2.6 at day 30 and 0.7 at day 90. Bricout et al. [5] measured the impact of HZ on pain using the visual analog scale and demonstrated a somewhat slower reduction of pain from rash onset; however, we believe our study to be the first in Italy to measure HZ-associated pain utilizing the widely used ZBPI questionnaire. We also demonstrated using the ZBPI questionnaire that the impact of HZ on activities of daily living such as sleep and mood was greater in the acute period.

To the best of our knowledge, this was the first study in Italy to measure the impact of HZ on QoL by using the EQ-5D to measure disutility. We estimated a mean disutility of 0.134 during the first month of the HZ episode (equivalent to QALY loss of 0.011 per month) using the recently published Italian time-trade-off values [13]. This corresponds to losing approximately 4.1 days of perfect health per month of HZ. We observed that the disutility increased (ie, worsened) with age. For patients without PHN, we estimated a mean disutility of 0.131 during the first month of the HZ episode.

It was clear that patients who developed PHN had a greater burden of pain from rash onset until at least day 120. The estimated mean utility for PHN patients for the period days 90–120 was 0.717, which appears to be less than the mean value of 0.798 estimated in all HZ patients from days 0 to 30. This is consistent with the known chronic effect of HZ on the quality of life of patients with PHN.

Our results also confirmed that HZ imposes a substantial economic burden on the health care system as a result of high medical costs related to medications, outpatient visits, and hospitalizations.

We calculated a mean direct medical cost per patient of €153 from the payer perspective and €297 from a societal perspective, which are similar to the mean cost from a payer perspective of €166 estimated in the 2005 retrospective study by Gialloretti et al. [7] and the mean cost of €136 excluding hospitalizations in the 2004 study based in Piemonte [6].

Costs associated with visits to GPs or specialists were low. Patients visited their GP an average of 2.2 times, and 62% of patients had more than 1 visit. Only 38 patients were referred to see a specialist. This is similar to the findings in Di Legami et al., in which a mean of 2.1 visits per case was reported [6].

A total of 7 patients were hospitalized, accounting for 31.5% of total direct medical costs from a payer perspective. This is less than the figure of 50.7% in Arpinelli et al. [16], as discussed in Panatto et al. [15]. It is possible that we have underestimated the frequency of hospitalizations as the study recruited only patients in the primary setting and may have excluded the proportion of hospitalized HZ patients who could have presented directly at the hospital rather than in an ambulatory health care setting. In addition, we may also have underestimated the costs of hospitalizations by estimating a mean duration of 7.8 days [14].

The biggest contribution to direct medical costs was medications. A total of 93.4% of all patients were prescribed antivirals. Approximately 63% of all patients were prescribed the more expensive antivirals, such as brivudine at a cost of €96 or valaciclovir at a cost of €60, whereas only 25% were prescribed the relatively less expensive aciclovir at a cost of €25. Medications accounted for approximately 49% of direct medical costs from a payer perspective, less than the 65.4% reported in the Italian study by Di Legami et al. [6] and somewhat higher than the figures for other countries (32%–47%), as discussed in the literature review by Panatto et al. [15].

The highest economic burden was observed in patients in the 50–59 years of age group, with a mean total cost of €515 per patient from a societal perspective, which is explained by the fact that missed work days associated with the HZ episode were only reported by patients under the age of 65 years. This finding agrees with the findings of Scott et al. [17], who concluded that being employed predicts higher societal costs and that the indirect costs of HZ are therefore associated with the age of onset of the disease. As expected, the lowest costs from a societal perspective were observed in the ≥80 age category (mean, €162 per patient).

Generally, there is a lack of data concerning the costs of complications other than PHN.

We calculated mean total costs of €286 and €592 from the payer and societal perspectives, respectively, for the 83 patients with non-PHN complications (cutaneous, neurological, and ocular), which were higher than the mean costs for patients with PHN. We calculated a mean of €319/€604 for patients with cutaneous complications, €174/€439 for patients with neurological complications, and €669/€1582 for patients with ocular complications from the payer/societal perspective. Few studies internationally have assessed costs by type of complication, and none in Italy. Recent studies in Japan [18] and Germany [19] have highlighted the high costs associated with complications, whereas Yawn et al. [20] demonstrated that complicated non-PHN HZ cases can cost more than PHN cases.

A potential limitation of the study is the relatively low population size of 44 000 persons across the regions under surveillance. Another limitation is that an unknown number of HZ cases may have been missed, in part due to patients not seeking medical care or patients seeking specialist dermatological (eg, hospital emergency) care without seeking primary care attention, and this could lead to an underestimation of the frequency of severe cases and the associated burden. However, the estimated overall incidence of HZ in Italian individuals aged ≥50 years is in line with those in this age group reported in a recent systematic review of European studies by Pinchinat et al. [21].

The strengths and limitations of this study in the context of HZ incidence are further discussed in another manuscript submitted elsewhere (unpublished).

Italy has the second highest proportion of worldwide elderly people, after Japan [22], making it likely that the burden of HZ disease will increase considerably. The Italian National Immunization Plan 2017–2019 recommended a vaccination program for all adults aged ≥65 years and all at-risk adults aged ≥50 years (such as adults with cardiovascular diseases, chronic obstructive pulmonary disease, diabetes, or those in need of immunosuppressive therapy). The planned objective is to vaccinate 20% of all adults ≥65 in 2018, 35% in 2019, and 50% in 2020.

## CONCLUSIONS

**Figure 3.**
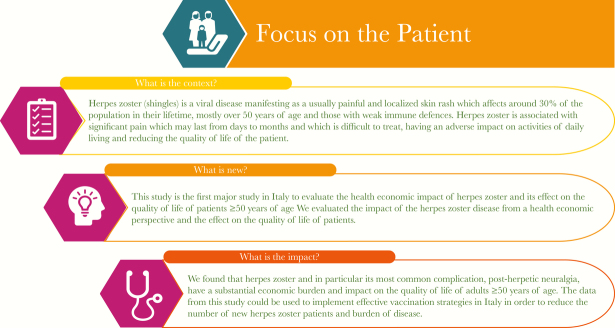
Focus on the patient.

Our study showed that HZ had a large economic burden and a substantial impact on the patients’ QoL and ability to function in their normal activities. The results documented the current clinical and economic burden of HZ and PHN in Italian adults aged 50 years and older. We have shown in this study that these vaccination objectives are of crucial importance to reduce the economic burden on society and the impact on QoL of the individual. Therefore, an appropriate preventive strategy, such as vaccination, is recommended to reduce the future burden. Figure 3 presents a summary of the context, outcomes, and impact of this study for health care providers.

## Supplementary Material

ofz007_suppl_supplementary_material

## References

[1] Bowsher D The lifetime occurrence of herpes zoster and prevalence of post-herpetic neuralgia: a retrospective survey in an elderly population. Eur J Pain 1999; 3:335–42. 10700361 10.1053/eujp.1999.0139

[2] Brisson M, Edmunds WJ, Law B, et al Epidemiology of varicella zoster virus infection in Canada and the United Kingdom. Epidemiol Infect 2001; 127:305–14. 11693508 10.1017/s0950268801005921 PMC2869750

[3] Edmunds WJ, Brisson M, Rose JD The epidemiology of herpes zoster and potential cost-effectiveness of vaccination in England and Wales. Vaccine 2001; 19:3076–90. 11312002 10.1016/s0264-410x(01)00044-5

[4] Cohen JI Herpes zoster. N Engl J Med 2013; 369:1766–7. 10.1056/NEJMc1310369 PMC4786186 24171531

[5] Bricout H, Perinetti E, Marchettini P, et al Burden of herpes zoster-associated chronic pain in Italian patients aged 50 years and over (2009-2010): a GP-based prospective cohort study. BMC Infect Dis 2014; 14:1–11. 25479617 10.1186/s12879-014-0637-6 PMC4268902

[6] Di Legami V, Gianino MM, Ciofi degli Atti M, et al; Zoster Study Group Epidemiology and costs of herpes zoster: background data to estimate the impact of vaccination. Vaccine 2007; 25:7598–604. 17889410 10.1016/j.vaccine.2007.07.049

[7] Gialloreti LE, Merito M, Pezzotti P, et al Epidemiology and economic burden of herpes zoster and post-herpetic neuralgia in Italy: a retrospective, population-based study. BMC Infect Dis 2010; 10:1–11. 20682044 10.1186/1471-2334-10-230 PMC2921387

[8] Levi M, Bellini I, Capecchi L, et al The burden of disease of herpes zoster in Tuscany. Hum Vaccin Immunother 2015; 11:185–91. 25483534 10.4161/hv.35859 PMC4514367

[9] Cocchio S, Baldovin T, Furlan P, et al Cross-sectional study on hospitalizations related to herpes zoster in an Italian region, 2008–2016. Aging Clin Exp Res. In press. 10.1007/s40520-018-0968-z 29766448

[10] Coplan PM, Schmader K, Nikas A, et al Development of a measure of the burden of pain due to herpes zoster and postherpetic neuralgia for prevention trials: adaptation of the Brief Pain Inventory. J Pain 2004; 5:344–56. 15336639 10.1016/j.jpain.2004.06.001

[11] EuroQol. Website of the EuroQol Group 2016 http://www.euroqol.org/. Accessed 12 July 2016.

[12] Rabin R, de Charro F EQ-5D: a measure of health status from the EuroQol Group. Ann Med 2001; 33:337–43. 11491192 10.3109/07853890109002087

[13] Scalone L, Cortesi PA, Ciampichini R, et al Italian population-based values of EQ-5D health states. Value Health 2013; 16:814–22. 23947975 10.1016/j.jval.2013.04.008

[14] Coretti S, Codella P, Romano F, et al Cost-effectiveness analysis of herpes zoster vaccination in Italian elderly persons. Int J Technol Assess Health Care 2016; 32:233–40. 27624398 10.1017/S0266462316000337

[15] Panatto D, Bragazzi NL, Rizzitelli E, et al Evaluation of the economic burden of herpes zoster (HZ) infection. Hum Vaccin Immunother 2015; 11:245–62. 25483704 10.4161/hv.36160 PMC4514227

[16] Arpinelli F, Bonzanini AC, Visonà G La gestione clinica ed i costi dell’Herpes Zoster in Italia. G Farmacoeconomia 2000; 3:98–103.

[17] Scott FT, Johnson RW, Leedham-Green M, et al The burden of herpes zoster: a prospective population based study. Vaccine 2006; 24:1308–14. 16352376 10.1016/j.vaccine.2005.09.026

[18] Nakamura H, Mizukami A, Adachi K, et al Economic burden of herpes zoster and post-herpetic neuralgia in adults 60 years of age or older: results from a prospective, physician practice-based cohort study in Kushiro, Japan. Drugs Real World Outcomes 2017; 4:187–98. 28988331 10.1007/s40801-017-0119-4 PMC5684048

[19] Schmidt-Ott R, Schutter U, Simon J, et al Incidence and costs of herpes zoster and postherpetic neuralgia in German adults aged ≥50 years: a prospective study. J Infect 2018; 76:475–82. 29428228 10.1016/j.jinf.2018.02.001

[20] Yawn BP, Gilden D The global epidemiology of herpes zoster. Neurology 2013; 81:928–30. 23999562 10.1212/WNL.0b013e3182a3516e PMC3885217

[21] Pinchinat S, Cebrián-Cuenca AM, Bricout H, Johnson RW Similar herpes zoster incidence across Europe: results from a systematic literature review. BMC Infect Dis 2013; 13:1–10. 23574765 10.1186/1471-2334-13-170 PMC3637114

[22] United Nations Population Division. World population prospects: the 2006 revision population database. http://www.un.org/esa/population/publications/wpp2006/wpp2006.htm. Accessed 18 November 2017.

