# Supplementary material for: The Economic Burden and Impact on Quality of Life of Herpes Zoster and Postherpetic Neuralgia in Individuals Aged 50 Years or Older in Italy
Source: Open Forum Infect Dis. 2019 Jan 12;6(2):ofz007. doi: 10.1093/ofid/ofz007 (PMC6377935; doi:10.1093/ofid/ofz007)
Supplement: ofz007_suppl_supplementary_material [file ofz007_suppl_supplementary_material.docx]

Supplementary Data

The Economic Burden And Impact On Quality Of Life Of Herpes Zoster And Postherpetic Neuralgia In Individuals Aged 50 Years Or Older In Italy

Sean Matthews, Antonio De Maria, Marco Passamonti, Giovanni Ristori, Idalba Loiacono, Anna Puggina, Desmond Curran

*Open Forum Infectious Diseases*

<https://doi.org/10.1093/ofid/ofz007>

# Supplementary material

# Supplementary text

# Supplementary Table 1. Details of visit window intervals

# Supplementary Table 2. Unit costs and their sources

# Supplementary Table 3. Most frequent medications used to treat the herpes zoster episode by age group and overall

# Supplementary Table 4. Total costs by type of complication and age group

# Supplementary Table 5. Frequency of non-postherpetic neuralgia (PHN) complications by age group

# Supplementary Table 6 ZBPI Activities of Daily Living (ADL) Score Over Time until Day 90

# Supplementary Table 7 Total costs(€) from a payer/societal perspective by gender, PHN status and age group

# Supplementary Figure 1. Mean zoster brief pain inventory (ZBPI) worst pain scores by postherpetic neuralgia (PHN) category and time

HZ: herpes zoster

# Supplementary Figure 2. Mean zoster brief pain inventory (ZBPI) individual activities of daily living component scores by time from day 0 until day 90

# Supplementary Figure 3. Societal costs by cost category

# Supplementary Materials References

# Supplementary text

## *Visit Windows*

According to the study design 'All patients were to be followed up until being assessed on day 90 after the initial visit'. The initial visit being the first time the subject presents at one of the study sites. The initial visit does not correspond to the date of rash onset so we define Day 0 as the first day of herpes zoster rash (henceforth defined as HZR Day 0). Consequently, all analysis of data over time was carried out using HZR Day 0 as the HZ episode start date. This is consistent with the analysis of Coplan^1^. Previous studies among patients with zoster pain have demonstrated that pain scores less than 3 occurring more than 30 days after rash onset are associated with minimal interference with either activities of daily living (ADL) or quality of life (QoL); thus, we assume that scores of 1 and 2 occurring more than 30 days after HZ rash onset were scored as 0. Questionnaires were classified into time windows depending on the date of assessment relative to HZR day 0. To be considered evaluable at week 0 a questionnaire must have been filled in on or within 7 days of HZR Day 0. Questionnaires at other time points were considered evaluable if they were completed within time windows as detailed in Supplementary Table 1.

Questionnaires without a date of assessment were to be considered not evaluable. Only one questionnaire per patient per time window was analysed. In case of multiple evaluable questionnaires in a time window, the values from the questionnaire which was closest to the scheduled assessment time would be used to represent that time window.

*Statistical analysis*

A repeated measures analysis of variance (ANOVA) model including terms for gender, age group and age group by time (window) interaction was fitted to the EQ-5D utility scores. The LS mean estimates for time by age group was obtained from the ANOVA model. The PROC MIXED procedure in SAS version 9.2 was used to carry out the ANOVA, with all terms fitted as fixed effects. An unstructured variance-covariance was assumed^2^.

This model was used to estimate the mean values of the EQ-5D scores in both HZ and PHN patients. An estimate statement within the PROC MIXED procedure in SAS was used to estimate the mean EQ-5D utility score in all HZ patients during the first 30 days.

## *ZBPI Questionnaire*

The ZBPI questionnaire was used to quantify HZ pain and discomfort and to measure the impact on ADL and health. The ZBPI questionnaire is was adapted from the Brief Pain Inventory to make it a HZ-specific measure of pain severity that captures pain and discomfort caused by HZ^1^. It uses an 11-point Likert scale (0–10) to rate HZ pain and discomfort for four dimensions (worst, least, and average during the past 24 hours and now) and HZ pain and discomfort–related interference with seven functional status and ADL items: general activity, mood, walking ability, work, relations with others, sleep, and enjoyment of life. The 7 questions included in the functional status and ADL are summarized into a single score by taking the mean of the 7 items. If a question was missing a response, the mean of the remaining 6 items was used.

## *EQ-5D Questionnaire*

Quality of Life was assessed by the standardized, generic EQ-5D questionnaire^3,4^, which is designed for self-completion by the respondents and widely used across many disease areas to assess health states or outcomes of interventions. It consists of five items addressing mobility, self-care, usual activity, pain/discomfort and anxiety/depression. For each item, there are three response categories; no problem, some problems and extreme problems. On the basis of the responses to these items, a summary weighted health utility score is calculated using country- specific weights derived from a sample of the general population (in this case Italian time-trade-off values were used^5^). The ‘worst health’ state imaginable corresponds to a utility score of -0.38 whereas 1.00 represents ‘perfect health’. Negative scores are interpreted as being in a state worse than death.

# Supplementary Table 1. Details of visit window intervals.

| **Time Window*** | **Timepoint** |
| --- | --- |
| Day 0 – Day 7 | Day 0 |
| Day 8 – Day 22 | Day 15 |
| Day 23 – Day 45 | Day 30 |
| Day 46 – Day 75 | Day 60 |
| Day 76 – Day 105 | Day 90 |
| Day 106 – Day 135 | Day 120 |
| Day 136 – Day 165 | Day 150 |
| Day 166 – Day 195 | Day 180 |
| Day 196 – Day 225 | Day 210 |
| Day 226 – Day 255 | Day 240 |
| >=Day 256 | Day 270 |

*Day is calculated as date of assessment – date of rash onset.

# Supplementary Table 2. Unit costs and their sources

| **Type of resource** | **Source and amount** |
| --- | --- |
| **Physician visits (generalist or specialist)** | - The cost of a general practitioner visit is estimated to be €11.14)^6^ - The unit cost of all specialist visits was €20.66. This was obtained from ^7^. Specialist visits are fully reimbursed by the National Health Service. |
| **Hospital visits** | In the absence of detailed information regarding length of hospital stay, an average of 7.8 days was assumed.^8^  The cost of a hospital stay associated with a herpes zoster (HZ) episode was estimated to be €2695.^9^ |
| **Medications** | Prescription Medications can be assigned to one of three categories or classes as follows:  **CLASS A:**  In Italy, under the reference price system, the national healthcare system reimburses the lowest price among the prices of off-patent pharmaceuticals of the same equivalent group (equal composition in active ingredients, same pharmaceutical form, method of administration, number of units and same unit dosage). When patients refuse the substitution of a medicine within the system for generic substitution and/or if the doctor prescribes a pharmaceutical product with a price higher than the reference price, the difference is paid by the patient.  **CLASS H (Hospital pharmacy dispensed):**  This category consists of medicines requiring specialist supervision and eligible for reimbursement only when used for in-patient care (hospital use only).  **CLASS C:**  This class consists of pharmaceutical products that require a prescription but are not reimbursed by the national healthcare system.  The Costs were obtained from ^10^ and ^11^  **Over The Counter (OTC) medication :**  Over The Counter (OTC) medication is not reimbursed by the national healthcare system and is paid 100% by the patient. In Italy OTC prices are assigned freely by each pharmacy. Estimates of costs for OTC medication will be obtained from ^12^ and ^13^ |
| **Procedures** | The unit cost of herpes zoster-related diagnostic or therapeutic procedures was obtained from (Nomenclatore tariffario prestazioni specialistica ambulatoriale, 2013)^7^  **Procedure Cost**  Blood collection for related tests €9.41  Skin scrapings or vesicular fluid sample collection for virology test €6.80  Dressing of the area with rash €42.50  Treatment administration – muscular €6.80  Treatment administration – intravenous €10.05  Treatment administration – subcutaneous €6.80 |
| **Work loss** | Work loss associated with the HZ episode was assessed by the number of working days lost by the patient and the caregiver. Working days lost were valued by multiplying the number of days recorded by the national average daily earnings as defined by the most recently available national labour statistics  An average daily salary of €109 was used.^14^  For manual workers the first 3 days of absence from work is paid by the company. The remaining days will be covered by the INSP (Istituto nazionale della previdenza sociale)  For office workers and middle management the company covers the entire cost of the absence due to illness. |
| **Transportation Costs** | The total cost of both private and public transport related to the HZ episode was calculated based on the total number of kilometres travelled. A unit cost per km was assigned to obtain total costs according to the following data:^15-18^  **Mode of Transport Cost**  Private Car^15^ 0.51€/km  Taxi^16^ 1.09€/km  Public Transport Bus / Train^18^ Urban : 3.29€/km  Non-Urban : 4.25€/km  Ambulance^19^ 205€ per completed journey |

# Supplementary Table 3. Most frequent medications used to treat the herpes zoster episode by age group and overall

|  | **50–59 YOA**  **N=87** | | **60–64 YOA**  **N=38** | | **65–69 YOA**  **N=88** | | **70–79 YOA**  **N=98** | | **≥80**  **YOA**  **N=80** | | **Overall**  **≥50 YOA N=391** | |
| --- | --- | --- | --- | --- | --- | --- | --- | --- | --- | --- | --- | --- |
| **ATC Level 2 – Preferred Term** | **n** | **%** | **n** | **%** | **n** | **%** | **n** | **%** | **n** | **%** | **n** | **%** |
| **Antivirals for Systemic use** | 80 | 92.0 | 38 | 100 | 80 | 90.9 | 90 | 91.8 | 77 | 96.3 | 365 | 93.4 |
| - Valaciclovir | 26 | 29.9 | 15 | 39.5 | 30 | 34.1 | 36 | 36.7 | 21 | 26.3 | 128 | 32.7 |
| - Brivudine | 22 | 25.3 | 8 | 21.1 | 26 | 29.5 | 29 | 29.6 | 33 | 41.3 | 118 | 30.2 |
| - Aciclovir | 24 | 27.6 | 14 | 36.8 | 24 | 27.3 | 22 | 22.4 | 16 | 20 | 100 | 25.6 |
| - Famciclovir | 9 | 10.3 | 1 | 2.6 | 1 | 1.1 | 6 | 6.1 | 7 | 8.8 | 24 | 6.1 |
| **Antibiotics and Chemotherapeutics for dermatological use** | 27 | 31.0 | 14 | 36.8 | 27 | 30.7 | 28 | 28.6 | 31 | 38.8 | 127 | 32.5 |
| - Aciclovir | 26 | 29.9 | 12 | 31.6 | 27 | 30.7 | 23 | 23.5 | 25 | 31.3 | 113 | 28.9 |
| **Antiepileptics** | 19 | 21.8 | 11 | 28.9 | 29 | 33 | 36 | 36.7 | 24 | 30 | 119 | 30.4 |
| - Pregabalin | 17 | 19.5 | 9 | 23.7 | 28 | 31.8 | 32 | 32.7 | 21 | 26.3 | 107 | 27.4 |
| **Aanalgesics** | 19 | 21.8 | 7 | 18.4 | 25 | 28.4 | 34 | 34.7 | 27 | 33.8 | 112 | 28.6 |
| - Paracetamol/Codeine | 10 | 11.5 | 1 | 2.6 | 11 | 12.5 | 15 | 15.3 | 7 | 8.8 | 44 | 11.3 |
| - Paracetamol | 7 | 8 | 3 | 7.9 | 9 | 10.2 | 14 | 14.3 | 13 | 16.3 | 46 | 11.8 |

ATC: anatomical therapeutic chemical; n: number of patients taking a medication; N: number of patients in the age category; YOA: years of age

# Supplementary Table 4 Total costs by type of complication and age group

|  | **Perspective** | **50–59 YOA**  **N=87** | | **60–64 YOA**  **N=38** | | **65–69 YOA**  **N=88** | | **70–79 YOA**  **N=98** | | **≥80**  **YOA N=80** | | **Overall**  **≥50 YOA N=391** | |
| --- | --- | --- | --- | --- | --- | --- | --- | --- | --- | --- | --- | --- | --- |
| Type of Complication |  | **n** | **Mean (€)** | **n** | **Mean (€)** | **n** | **Mean (€)** | **n** | **Mean (€)** | **n** | **Mean (€)** | **n** | **Mean (€)** |
| Cutaneous | HCS | 7 | 109 | 4 | 803 | 11 | 345 | 13 | 372 | 7 | 109 | 42 | 319 |
|  | Soc | 7 | 1328 | 4 | 863 | 11 | 447 | 13 | 481 | 7 | 210 | 42 | 604 |
| Neurological | HCS | 7 | 114 | 9 | 102 | 12 | 165 | 17 | 286 | 13 | 120 | 58 | 174 |
|  | Soc | 7 | 939 | 9 | 782 | 12 | 220 | 17 | 392 | 13 | 194 | 58 | 439 |
| Ocular | HCS | 2 | 151 | 1 | 119 | 6 | 1018 | 4 | 804 | 2 | 148 | 15 | 669 |
|  | Soc | 2 | 3560 | 1 | 5867 | 6 | 1146 | 4 | 848 | 2 | 241 | 15 | 1582 |
| Any non-PHN complication | HCS | 13 | 110 | 10 | 373 | 22 | 383 | 25 | 368 | 18 | 115 | 88 | 283 |
|  | Soc | 13 | 1214 | 10 | 990 | 22 | 463 | 25 | 475 | 18 | 196 | 88 | 583 |

PHN: postherpetic neuralgia; HCS: health care system; n: number of patients taking a medication; N: number of patients in the age category; Soc: societal; YOA: years of age; €: euro currency

# Supplementary Table 5 ZBPI Worst Pain Score over time until Day 90 by PHN status

| **Day** |  | **All Patients**  **(N=391)** | **PHN**  **Patients**  **(N=40)** | **HZ only**  **(No PHN)**  **(N=351)** |
| --- | --- | --- | --- | --- |
| Day 0 | N | 361 | 35 | 326 |
|  | Mean | 5.7 | 7.8 | 5.5 |
|  | SD | 2.63 | 2.18 | 2.59 |
|  | Median | 6.0 | 8.0 | 6.0 |
| Day 15 | N | 368 | 39 | 329 |
|  | Mean | 4.2 | 7.5 | 3.8 |
|  | SD | 2.94 | 1.45 | 2.81 |
|  | Median | 4.0 | 7.0 | 4.0 |
| Day 30 | N | 355 | 38 | 317 |
|  | Mean | 2.6 | 7.3 | 2.1 |
|  | SD | 3.04 | 1.95 | 2.64 |
|  | Median | 0.0 | 7.0 | 0.0 |
| Day 60 | N | 360 | 39 | 321 |
|  | Mean | 1.3 | 5.6 | 0.8 |
|  | SD | 2.35 | 2.46 | 1.72 |
|  | Median | 0.0 | 6.0 | 0.0 |
| Day 90 | N | 357 | 40 | 317 |
|  | Mean | 0.7 | 5.7 | 0.0 |
|  | SD | 1.94 | 1.96 | 0.33 |
|  | Median | 0.0 | 5.5 | 0.0 |

PHN: postherpetic neuralgia; HZ Only: Patients who did not develop PHN; SD: Standard Deviation

# Supplementary Table 6 ZBPI Activities of Daily Living (ADL) Score Over Time until Day 90

|  |  | | | | | | |
| --- | --- | --- | --- | --- | --- | --- | --- |
| **Time Point** |  | **50 - 59  YOA  N = 87** | **60 - 64  YOA  N = 38** | **65 - 69  YOA  N = 88** | **70 - 79  YOA  N = 98** | **≥ 80  YOA  N = 80** | **Total   N = 391** |
| Day 0 | N | 76 | 34 | 82 | 89 | 76 | 357 |
|  | Mean | 3.327 | 2.042 | 3.038 | 3.693 | 4.492 | 3.478 |
|  | SD | 2.687 | 2.030 | 2.644 | 2.576 | 2.856 | 2.707 |
|  | Median | 2.86 | 1.50 | 2.71 | 3.29 | 4.71 | 3.00 |
| Day 15 | N | 77 | 38 | 82 | 94 | 77 | 368 |
|  | Mean | 2.160 | 1.588 | 2.688 | 3.060 | 3.562 | 2.742 |
|  | SD | 2.532 | 1.812 | 2.689 | 2.760 | 3.102 | 2.749 |
|  | Median | 1.00 | 0.93 | 2.21 | 2.14 | 3.43 | 1.86 |
| Day 30 | N | 75 | 33 | 80 | 89 | 77 | 354 |
|  | Mean | 1.208 | 0.835 | 2.076 | 2.029 | 2.345 | 1.823 |
|  | SD | 2.139 | 1.639 | 2.607 | 2.485 | 2.665 | 2.459 |
|  | Median | 0.00 | 0.00 | 1.00 | 1.00 | 1.29 | 0.43 |
| Day 60 | N | 75 | 35 | 83 | 92 | 75 | 360 |
|  | Mean | 0.708 | 0.380 | 1.005 | 1.186 | 1.470 | 1.026 |
|  | SD | 1.695 | 1.526 | 1.729 | 1.956 | 2.367 | 1.931 |
|  | Median | 0.00 | 0.00 | 0.00 | 0.00 | 0.00 | 0.00 |
| Day 90 | N | 76 | 35 | 81 | 91 | 74 | 357 |
|  | Mean | 0.385 | 0.253 | 0.635 | 0.656 | 0.770 | 0.578 |
|  | SD | 1.154 | 1.163 | 1.584 | 1.570 | 1.622 | 1.471 |
|  | Median | 0.00 | 0.00 | 0.00 | 0.00 | 0.00 | 0.00 |

YOA: years of age; SD: Standard deviation

# Supplementary Table 7 Total costs(€) from a payer/societal perspective by gender, PHN status and age group

|  | |  | | | | | | |
| --- | --- | --- | --- | --- | --- | --- | --- | --- |
| **Category** | **Perspective** |  | **50 - 59  YOA** | **60 - 64  YOA** | **65 - 69  YOA** | **70 - 79  YOA** | **≥ 80  YOA** | **Total** |
| Male | HCS | N | 22 | 20 | 38 | 43 | 31 | 154 |
|  |  | Mean | 116 | 81 | 106 | 209 | 105 | 133 |
|  |  | SD | 73 | 42 | 109 | 434 | 45 | 242 |
|  |  | Median | 107 | 77 | 98 | 110 | 118 | 107 |
|  | SOC | N | 22 | 20 | 38 | 43 | 31 | 154 |
|  |  | Mean | 246 | 130 | 146 | 265 | 148 | 192 |
|  |  | SD | 303 | 92 | 128 | 456 | 77 | 281 |
|  |  | Median | 137 | 121 | 121 | 132 | 128 | 126 |
| Female | HCS | N | 65 | 18 | 50 | 55 | 49 | 237 |
|  |  | Mean | 130 | 232 | 208 | 204 | 105 | 166 |
|  |  | SD | 338 | 646 | 532 | 524 | 48 | 431 |
|  |  | Median | 82 | 71 | 103 | 89 | 107 | 96 |
|  | SOC | N | 65 | 18 | 50 | 55 | 49 | 237 |
|  |  | Mean | 606 | 585 | 252 | 289 | 170 | 366 |
|  |  | SD | 1347 | 1469 | 562 | 557 | 95 | 904 |
|  |  | Median | 116 | 115 | 125 | 137 | 146 | 129 |
| All Patients | HCS | N | 87 | 38 | 88 | 98 | 80 | 391 |
|  |  | Mean | 126 | 153 | 164 | 206 | 105 | 153 |
|  |  | SD | 294 | 445 | 408 | 485 | 46 | 368 |
|  |  | Median | 93 | 71 | 101 | 106 | 112 | 103 |
|  | SOC | N | 87 | 38 | 88 | 98 | 80 | 391 |
|  |  | Mean | 515 | 346 | 206 | 278 | 162 | 297 |
|  |  | SD | 1182 | 1024 | 433 | 512 | 89 | 730 |
|  |  | Median | 119 | 120 | 121 | 136 | 137 | 128 |
| PHN | HCS | N | 6 | 1 | 9 | 12 | 12 | 40 |
|  |  | Mean | 94 | 117 | 128 | 299 | 135 | 176 |
|  |  | SD | 48 | . | 50 | 286 | 66 | 179 |
|  |  | Median | 83 | 117 | 160 | 138 | 142 | 126 |
|  | SOC | N | 6 | 1 | 9 | 12 | 12 | 40 |
|  |  | Mean | 1164 | 186 | 207 | 429 | 239 | 426 |
|  |  | SD | 1486 | . | 74 | 388 | 137 | 663 |
|  |  | Median | 386 | 186 | 244 | 230 | 229 | 230 |
| HZ with Complications, no PHN | HCS | N | 15 | 10 | 19 | 24 | 15 | 83 |
|  |  | Mean | 107 | 373 | 425 | 359 | 114 | 286 |
|  |  | SD | 59 | 859 | 839 | 767 | 33 | 648 |
|  |  | Median | 95 | 126 | 122 | 115 | 117 | 116 |
|  | SOC | N | 15 | 10 | 19 | 24 | 15 | 83 |
|  |  | Mean | 1067 | 990 | 500 | 459 | 181 | 592 |
|  |  | SD | 2187 | 1915 | 877 | 772 | 67 | 1286 |
|  |  | Median | 153 | 178 | 159 | 205 | 175 | 172 |

PHN: postherpetic neuralgia; HCS: health care system; n: number of patients taking a medication; N: number of patients in the age category; Soc: societal; YOA: years of age; €: euro currency

# Supplementary Figure 1. Mean zoster brief pain inventory (ZBPI) worst pain scores by postherpetic neuralgia (PHN) category and time

# Supplementary Figure 2. Mean zoster brief pain inventory (ZBPI) individual activities of daily living component scores by time from day 0 until day 90

# Supplementary Figure 3. Societal costs by cost category

**
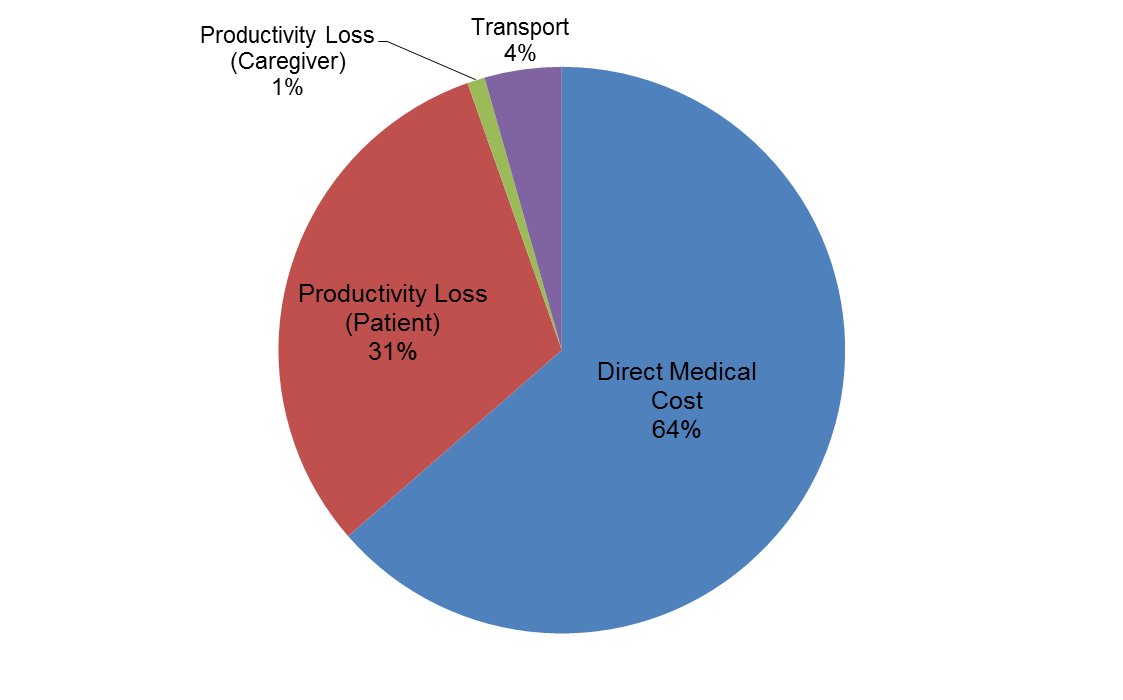
**

# Supplementary Materials References

1. Coplan PM, Schmader K, Nikas A, et al. Development of a measure of the burden of pain due to herpes zoster and postherpetic neuralgia for prevention trials: adaptation of the brief pain inventory. *J Pain.* Aug 2004;5(6):344-356.

2. Verbeke GM, G. *Linear mixed models for longitudinal data.* Springer-Verlag New York; 2000.

3. EuroQol, 2016. Website of the EuroQol group. http://www.euroqol.org/ Accessed 12 Jul 2016.

4. Rabin R, de Charro F. EQ-5D: a measure of health status from the EuroQol Group. *Ann Med.* Jul 2001;33(5):337-343.

5. Scalone L, Cortesi PA, Ciampichini R, et al. Italian population-based values of EQ-5D health states. *Value Health.* Jul-Aug 2013;16(5):814-822.

6. Gialloreti LE, Merito M, Pezzotti P, et al. Epidemiology and economic burden of herpes zoster and post-herpetic neuralgia in Italy: a retrospective, population-based study. *BMC Infect Dis.* Aug 3 2010;10:230.

7. Nomenclatore tariffario prestazioni specialistica ambulatoriale. (2013). Retrieved from http://www.salute.gov.it/portale/temi/p2_6.jsp?id=1767&area=programmazioneSanitariaLea&menu=lea.

8. Coretti S, Codella P, Romano F, Ruggeri M, Cicchetti A. Cost-Effectiveness Analysis of Herpes Zoster Vaccination in Italian Elderly Persons. *Int J Technol Assess Health Care.* Jan 2016;32(4):233-240.

9. Panatto D, Bragazzi NL, Rizzitelli E, et al. Evaluation of the economic burden of Herpes Zoster (HZ) infection. *Hum Vaccin Immunother.* 2015;11(1):245-262.

10. Tabelle Farmaci di classe A e H. (2016). Retrieved 2016, from Agenzia Italiana del farmaco: http://www.aifa.gov.it/content/tabelle-farmaci-di-classe-e-h-al-15092016.

11. Federazione nazionale unitaria titolari di farmacia. (2017). Retrieved May 2017, from Federazione nazionale unitaria titolari di farmacia: federfarma.it.

12. Starbene. (2017). Retrieved 2017, from Starbene: www.starbene.it.

13. efarma. (2017). Retrieved 2017, from efarma: http://www.efarma.it.

14. ISTAT. (n.d.). Retrieved 2017, from IL MERCATO DEL LAVORO: https://www.istat.it/it/files/2017/03/Mercato-del-lavoro-IV-trim-2016.pdf?title=Il+mercato+del+lavoro+-+10%2Fmar%2F2017+-+Testo+integrale+e+nota+metodologica.pdf.

15. Automobile Club d'Italia. (n.d.). Retrieved 2017, from http://www.aci.it.

16. Comune di milano. (n.d.). Retrieved 2017, from Comune di milano: http://www.comune.milano.it/wps/portal/ist/it/servizi/mobilita/taxi/tariffe.

17. Ministero della Salute. Progetto Mattoni SSN. Pronto Soccorso e sistema 118. Proposta metodologica per la valutazione dei costi dell’emergenza.

18. Della Porta AG, A. *La riforma del trasporto pubblico locale in Italia nella prospettiva aziendale. Il difficile compromesso tra economicità aziendale ed efficacia sociale,. In A. G. A. Della Porta, La riforma del trasporto pubblico locale in Italia nella prospettiva aziendale. Il difficile compromesso tra economicità aziendale ed efficacia sociale, (p. 221). Milan: FrancoAngeli.* 2013.

19. Veronese G, Marchesini G, Forlani G, et al. Costs associated with emergency care and hospitalization for severe hypoglycemia. *Nutr Metab Cardiovasc Dis.* Apr 2016;26(4):345-351.
